# ^13^C NMR as a foundation for machine learning models of polysaccharides

**DOI:** 10.1063/4.0001213

**Published:** 2026-06-15

**Authors:** Stephanann M. Costello, Zeina C. Sleiman, John N. Glushka, Breeanna R. Urbanowicz, Arthur S. Edison

**Affiliations:** 1 Department of Biochemistry and Molecular Biology, University of Georgia, Athens, Georgia 30602, USA; 2 Complex Carbohydrate Research Center, University of Georgia, Athens, Georgia 30602, USA

## Abstract

Polysaccharide structural assignment via nuclear magnetic resonance (NMR) spectroscopy remains an analytical challenge due to spectral overlap because of limited chemical shift dispersion. This challenge is exacerbated by the wide-spread use of proton (^1^H) detection. Progress has also been hindered by the dispersed nature of carbohydrate databases and the restricted applicability of most prediction tools, which provide limited atom-specific residue discrimination in larger polymers. These limitations hamper the efficient characterization of glycosyltransferase (GT) activity, which depends on defining donor–acceptor substrate pairs and accurately identifying corresponding products. Here, we demonstrate the utility of high field ^13^C-detected NMR for the assignment of two homohexamers. Laminarihexaose and xylohexaose were analyzed using 2D heteronuclear correlation and correlation via long-range coupling (COLOC) experiments to evaluate whether complete residue level assignments could be achieved. The COLOC experiment revealed long range correlations that were not resolved using heteronuclear multiple bond correlation, and the 1D ^13^C spectra provided exceptional resolution, including distinct shoulders corresponding to residue specific chemical shift differences previously assumed to be indistinguishable. These findings suggest that high field ^13^C NMR can provide the nuanced atom level information required to train machine learning models for predicting chemical shift assignments of large, complex, and highly degenerate polysaccharides. Such models offer a promising framework for rapid structural identification of GT reaction products, enabling progress of high throughput characterization of plant derived polysaccharides central to renewable biomaterial development.

## I. INTRODUCTION

Carbohydrates are ubiquitous to all kingdoms of life, serving invaluable structural and functional roles in diverse cell types and viruses. Among all organisms, plants produce some of the most diverse, abundant, and complex carbohydrates found in Nature. These molecules comprise major components of the plant cell wall, act as key energy reserves, and function as important mediators of plant defense. From an economic perspective, polysaccharides are indispensable in nutrition, pharmaceuticals, and industrial applications.^1^ Despite this utility, plant-derived carbohydrates remain an underutilized source of feedstocks for renewable biofuels and biomaterials.

The valorization of plant biomass, including industrial and agricultural plant waste, for renewable biomaterials requires a detailed understanding of the complex carbohydrates produced by these organisms. Therefore, cataloging the cellular machinery responsible for synthesizing this diversity of carbohydrates is essential. Enzymes that catalyze glycosidic bond formation, called glycosyltransferases (GTs), are of particular interest for research in plant polysaccharides. GTs operate in a concerted fashion, either through an inverting or retaining mechanism to form a new glycosidic bond, thereby elongating a linear polysaccharide chain or functioning in side chain or glycosyl substituent addition. To fully elucidate the functional relationship of these enzymes *in vitro* and better understand the large macromolecular structures they assemble, each GT requires individual characterization. From a structural perspective, there are three key questions that frame a GT's cellular role in product formation: (1) whether a specific donor–acceptor substrate pair supports glycosidic bond formation, (2) what the linkage configuration is (stereospecificity), and (3) to which acceptor-residue the sugar is added (regiospecificity).

Nuclear magnetic resonance (NMR) spectroscopy is an invaluable analytical tool for studying glycan structure. Extensive advances in methodology, instrumentation, and data analysis have already significantly improved spectral analysis and structural assignment of these complex molecules.^2^ Nevertheless, carbohydrate structural assignments remain difficult, with many limitations arising due to peak overlap and minimal chemical shift dispersion—a complexity that increases with each additional monomeric unit of a polysaccharide. In a proton (^1^H) NMR spectrum, most carbohydrate resonances span a narrow window of approximately 3.2–6.0 ppm, with anomeric protons resonating at frequencies between ∼4.4 and 6.0 ppm. Consequentially, anomeric signals span the water region (∼4.7 ppm), which gives rise to its own host of considerations, while the remaining non-anomeric signals are compressed into a spectral width of less than 3 ppm. Combined with strong coupling effects, this leads to severe and complex spectral overlap of features, hindering residue-specific assignments. Implementation of two-dimensional (2D) homo- and heteronuclear NMR experiments aid in reducing spectral overlap. Moreover, the 2D ^1^H-detected heteronuclear multiple bond correlation (HMBC) is the primary experiment to obtain correlations across the glycosidic bond for sequence-specific assignments.

^13^C NMR offers substantially greater chemical shift dispersion than ^1^H NMR,^3^ with most carbohydrate resonances spanning ∼50 to 90 ppm, and ∼90 to 110 ppm for anomeric signals. Additionally, at natural abundance, homonuclear J couplings are negligible, leading to single narrow peaks. The 2D heteronuclear correlation (^13^C,^1^H-HETCOR or HETCOR) experiment capitalizes on the digital resolution of the directly observed ^13^C dimension. However, due to sensitivity-limitations of ^13^C NMR, HETCOR has largely been replaced by the ^1^H-detected heteronuclear single quantum correlation experiment (^1^H,^13^C-HSQC or HSQC). Therefore, the classic suite of 2D experiments for sequentially assigning the chemical shifts of carbohydrates, along with their anomeric configuration, usually rely upon multiple ^1^H-detected 2D experiments.^2^ By detecting ^1^H in heteronuclear experiments, the large ^13^C chemical shift range is poorly digitized in the indirect dimension and requires substantial increases in measurement time to improve the resolution. Presently, the advantages of ^13^C direct detection are more extensively leveraged in protein NMR spectroscopy,^4,5^ a foundation in macromolecular structural elucidation that could translate to polysaccharides. Indeed, many modern-day methods pay homage to the substantial historical emphasis placed on ^13^C detection methods. Notable examples include the seminal work of John L. Markley, which demonstrated near-complete residue spin system characterization of a protein from a single 2D ^13^C experiment,^6^ and the systematic compilation of ^13^C chemical shift data for carbohydrates, which continues to underpin contemporary analyses.^7^

Among the earlier advances in ^13^C detection is the ^13^C-detected equivalent of the HMBC—the correlation via long range coupling (COLOC) experiment—published in 1984 by Kessler *et al.*,^8^ with variations developed in subsequent years.^9–11^ A narrow PubMed search query with the terms “NMR” and “COLOC” returns 44 publications that span 1986–2026, with only 2 since 2006. Among these recent publications, the COLOC experiment was used to aid in the structural assignment of an aromatic heterocyclic compound,^12^ and a newly isolated isoflavone glucoside.^13^ Prior applications typically employed the COLOC experiment to define the sequence of small glycans consisting of 2–3 residues, often with distinct sugar moieties.^14^ The primary uses for COLOC have been in natural product chemistry, aiding in the structural elucidation of terpene and phenolic phytochemicals, typically with instrument field-strengths of ≤14.1 T (600 MHz ^1^H frequency).^15^ Although the usage of the COLOC experiment as described has largely been replaced by HMBC, the concept of long-range heteronuclear correlations in NMR has steadily advanced. This framework has proven valuable for small-molecule metabolomics^16^ and has a considerable history in protein structural characterization,^5,17,18^ showcasing the advantage of direct heteronuclear detection to overcome solvent interference, dynamic range, and densely clustered spectra, particularly with modern high field magnets (≥1.0 GHz).

With the advent of high field magnets, cryogenically cooled and carbon tuned probes, together with the rapid development of machine learning (ML) and artificial intelligence (AI), the time is ripe for developing improved experimentally based computational models for complex glycan and polysaccharide assignments. Currently, databases for carbohydrates are dispersed and highly variable in the information they contain. The Biological Magnetic Resonance Data Bank (BMRB) is the primary international, open-access archive for NMR-derived data^19^ and contains many carbohydrate entries but often lists chemically degenerate shifts across multiple atoms. Carbohydrate-specific tools, such as CASPER (Computer Assisted Spectrum Evaluation of Regular polysaccharides),^20–23^ CSDB (Carbohydrate Structural DataBase),^24–26^ and GlyNest,^27^ have made great strides in assigning and predicting mono- to tetra-saccharides and larger heterogenous polysaccharide chemical shifts through the curation of their own reference datasets. However, the accurate prediction and assignment of larger (i.e., degree of polymerization > 4), structurally degenerate polysaccharides, which are relevant to plants and the characterization of the GTs that synthesize their cell walls, remains a challenging and largely unsolved problem. Therefore, to demonstrate a method to increase the threshold of identification of longer polymers, two homohexamers were chosen for this study: laminarihexaose and xylohexaose. These homohexamers are ideal due to their simple representation of plant carbohydrates. Xylohexaose is a pentose-based oligosaccharide consisting of six β(1 → 4) linked xylosyl residues, while laminarihexaose is hexose-based with a structure consisting of six β(1 → 3) linked glucosyl residues. Many plant carbohydrates are a challenge to breakdown due to their heterogeneity and, thus, are currently wasted in most industrial applications. Improving the structural understanding of polysaccharide building blocks will help research in the development of new valorization strategies.^28^

Here, we propose the use of high-field ^13^C NMR spectroscopy as the path toward improving NMR glycan experimental databases and implementing future ML/AI assignment algorithms for large and/or degenerate polysaccharides. The two homohexamers, laminarihexaose and xylohexaose, were used to demonstrate the utility of a 25.8 T (1.1 GHz) NMR spectrometer equipped with a 5-mm TXO ^13^C/^15^N-optimized cryoprobe. We show that this technology significantly improved resolution and revealed the small chemical shift differences in these repeating residues. Furthermore, we show that 2D COLOC and HETCOR experiments can provide the necessary correlations across the glycosidic bonds to sequentially assign many of the resonances in these polysaccharides. It is envisioned that a ML model designed and trained with 1D ^13^C spectra obtained from a large variety of known polysaccharides with varying structures would be able to predict the nuanced chemical shifts of these degenerate species. Thus, 2D COLOC and HETCOR data can then serve as orthogonal validation tools within a design–build–test–learn framework, ultimately enabling a high‐throughput workflow for structural assignment of plant polysaccharides and for characterizing the products of glycoenzymes.

## II. METHODS

### A. Preparation of carbohydrate samples and NMR data acquisition

Samples of natural abundance ^13^C sucrose (200 mM, J. T. Baker CAS # 57-50-1), laminarihexaose (50 mM, Megazyme, SKU 700004963, 29842-30-6), and xylohexaose (∼50 mM, AmBeed, SKU A1144690, CAS #: 49694-21-5) were prepared in 100% D_2_O with 10 mM DSS (sodium 2,2-dimethyl- 2-silapentane-5-sulfonate) added as a chemical shift reference. All three samples were dissolved in 180 *μ*l and loaded into 3 mm NMR tubes (Bruker).

Data were collected on a Bruker Avance Neo 1.1 GHz NMR equipped with a 5 mm TXO cryoprobe for ^13^C observed experiments, a 3 mm TCI cryoprobe for ^1^H observed 2D NMR experiments, and a Bruker SampleJet sample changer. Standard Bruker pulse programs were used to collect the core suite of NMR data for each sample and consisted of 1D ^1^H, ^13^C, 2D HSQC, HMBC, HETCOR, HETCOR-TOCSY, COLOC, and DQF COSY. Detailed parameter settings and any deviations from the standard Bruker pulse programs are reported in supplementary material Table I and are available with the data on the NAN resource connector. Topspin 4.5.0 (Bruker) was used to operate the spectrometer.

### B. Data processing and analysis

All NMR spectra were processed in TopSpin 4.5.0, with detailed processing parameters in supplementary material Table II.

NMRViewJ (v 9.2.0-b27 with Java 1.8.0_192 amd64, One Moon Scientific, Inc., Westfield, NJ, USA)^29,30^ was used to visualize data and map correlations across the HETCOR and COLOC spectra. PDF (portable document format) exports of each NMRViewJ window were used to make the figures representing the data. Structural assignment of sucrose and the hexamers are demonstrated with the 1D ^13^C, HETCOR, HETCOR-TOCSY, and COLOC NMR spectra. The high resolution HMBC and DQF COSY were evaluated through the lens of providing secondary confirmation of the assignments. CASPER simulations were also generated for xylohexaose and laminarihexaose (see the supplementary material) as a reference to aid in the assignment of resonances.

## III. RESULTS

### A. 1D ^1^H vs ^13^C spectra

**FIG. 1.**
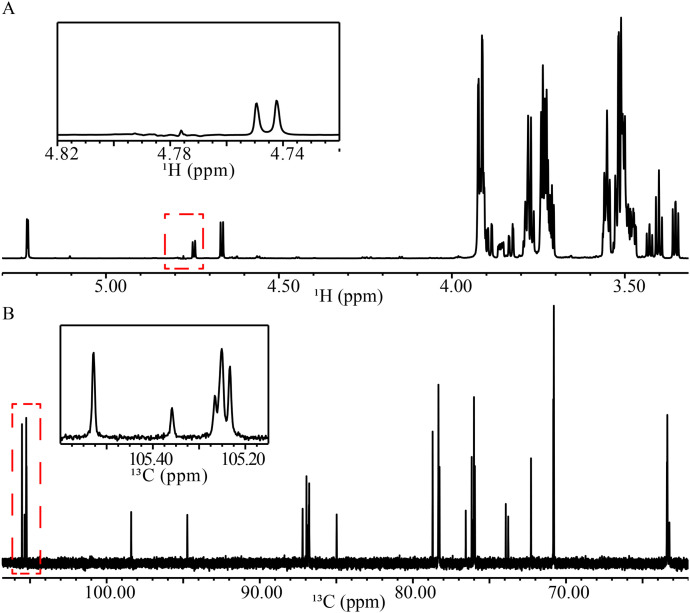
1.1 GHz 1D ^1^H (a) and ^13^C (b) spectra of laminarihexaose. Expansions show the more overlapped or troublesome anomeric regions. The anomeric ^1^H peaks are attenuated during water suppression, whereas five distinct peaks are visible in the tightly clustered ^13^C anomeric region.

Laminarihexaose (Fig. 1) is a repeating β(1→3) linked glucose chain with an anomeric reducing end that is in equilibrium between α/β configurations. Thus, 7 anomeric resonances are expected in both the 1D ^1^H and ^13^C spectra. Considering the remaining atoms, the ^1^H spectrum should contain a total of 42–49 resonances, depending on how many are sensitive to anomers from the reducing end. In contrast, the total number of ^13^C signals were anticipated to be between 36 and 42. Save the anomeric signals, all other ^1^H resonances in laminarihexaose occur between 3.3 and 4.0 ppm [Fig. 1(a)], making it essentially impossible to resolve this region. In the anomeric region, four laminarihexaose ^1^H resonances are obscured or eliminated by the residual water signal [Fig. 1(a) insert].

In contrast, 35 peaks were visible in the 1D ^13^C NMR spectrum for laminarihexaose [Fig. 1(b), supplementary material Table III], including all seven anomeric carbons, some of which had nearly degenerate chemical shifts [Fig. 1(b) inset]. All 1D ^13^C resonances in sucrose were resolved [supplementary material Figs. 1(a) and 1(b)], and the majority of ^13^C resonances were resolved in xylohexaose [supplementary material Figs. 1(c) and 1(d)]. These results are summarized and compared to ^1^H in supplementary material Table III.

### B. Advantages of ^13^C detection in 2D correlation experiments

#### 1. HSQC vs HETCOR

**FIG. 2.**
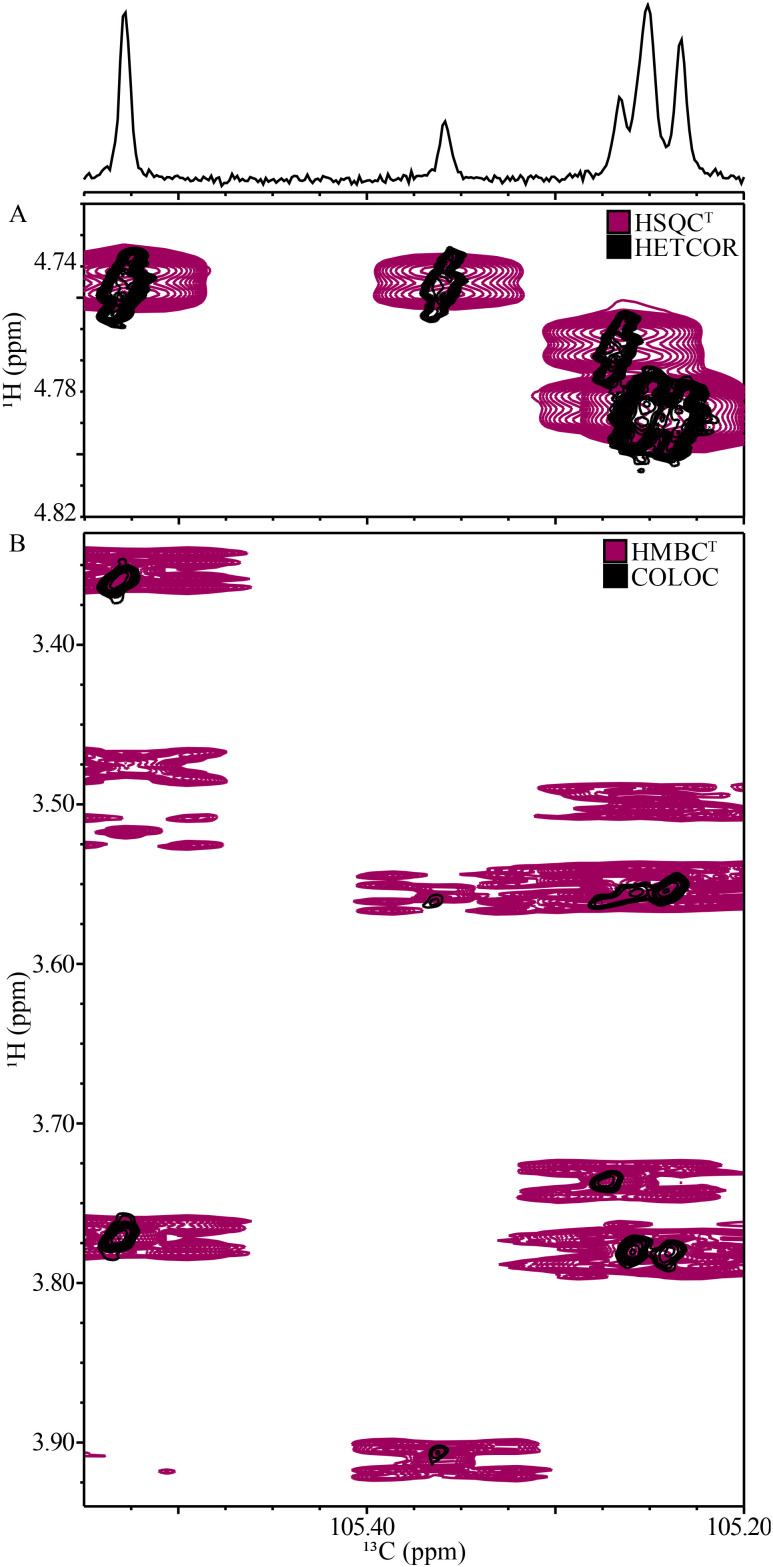
Comparison of anomeric peaks in laminarihexaose resolved by HSQC vs HETCOR (a) and HMBC vs COLOC (b). The ^1^H observed HSQC and HMBC axes were transposed (^T^) to match the ^13^C observed experiments. Both ^13^C detected experiments offer improved resolution for assigning the sequence of laminarihexaose, with major advantages seen in both the HETCOR and COLOC for the three peaks in the 1D that tightly cluster between 105.20 and 105.30 ppm.

HSQC and HETCOR provide similar ^1^J_CH_ correlations, but HSQC detects ^1^H and HETCOR detects ^13^C (supplementary material Fig. 2). The two main advantages enabled by HETCOR are (1) very high ^13^C resolution in the direct dimension and (2) avoidance of residual water or ^1^H-containing buffers. Figure 2(a) provides a comparison of transposed HSQC (HSQC^T^) and HETCOR data for the anomeric expansions of laminarihexaose shown in Fig. 1. Both experiments provide similar data, but the HSQC does not have enough ^13^C resolution to distinguish all the peaks. In addition to limited ^13^C resolution, the HSQC has limited ^1^H resolution due to technical limitations of decoupling ^13^C during ^1^H acquisition with a cryogenic probe. In contrast, the HETCOR experiment can be recorded with a long acquisition time that has no ^1^H decoupling limitation, providing extremely high-resolution ^13^C data. The HETCOR experiment resolved all five anomeric signals predicted for that region. Similar trends can be seen in the xylohexaose data (supplementary material Fig. 3).

#### 2. HMBC vs COLOC

**FIG. 3.**
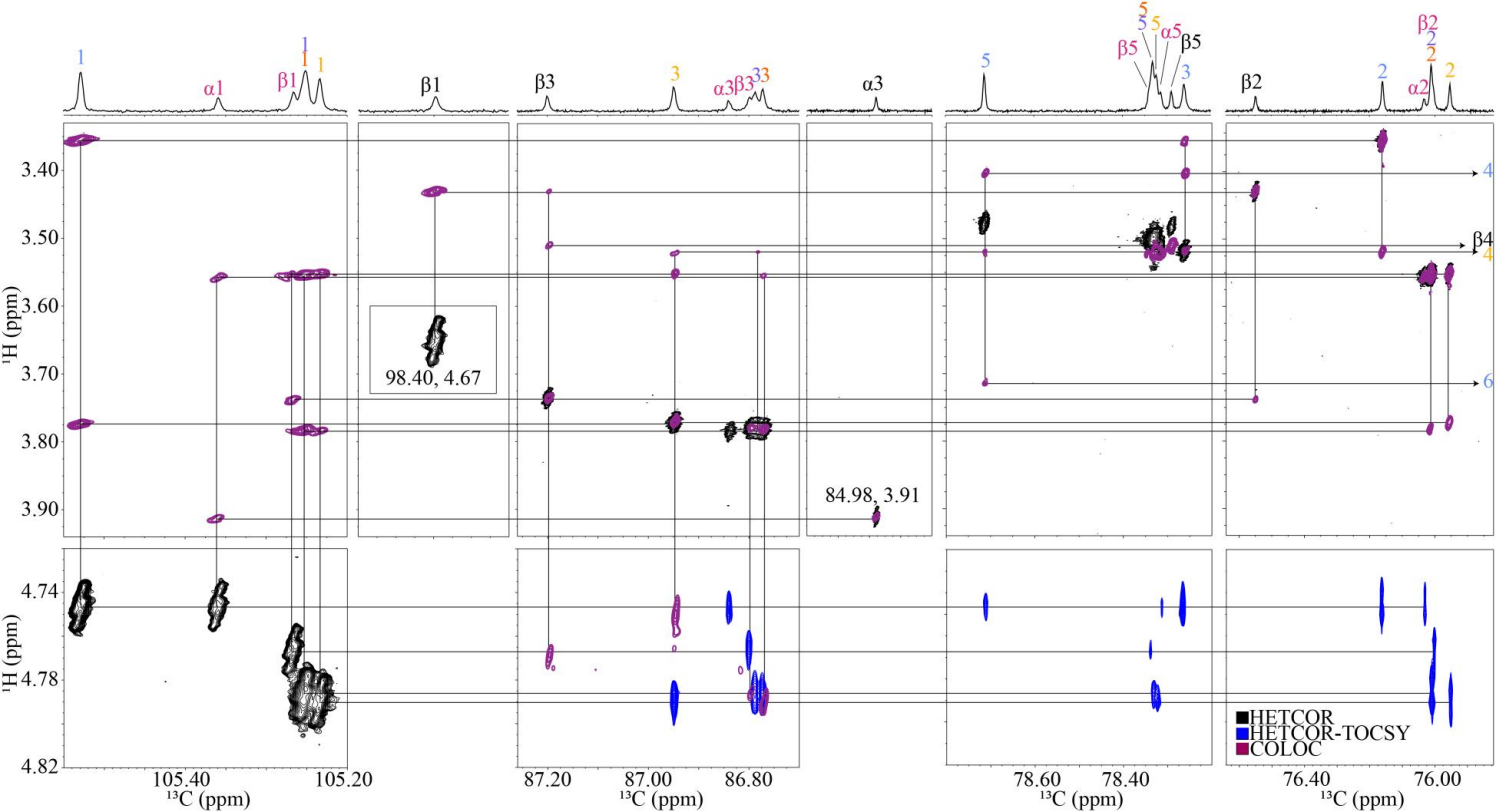
2D NMR data demonstrating the sequence assignment of laminarihexaose. HETCOR (black), HETCOR-TOCSY (blue), and COLOC (magenta) spectral regions that contain correlations across the glycosidic bond (C/H_1_ → C/H_3_). Correlations to C/H_2_ and C/H_5_ are also shown. Regions that include correlations for atoms 4 and 6 are omitted here, though correlations that extend to these regions are represented by arrows pointing to their associated atom. See the supplementary material Fig. 7 for all spectral regions. The intra-residue correlations in the HETCOR-TOCSY for the anomeric peaks are included as they were particularly useful in providing added clarity to the original HETCOR peaks. Single bond correlations for the anomeric peaks are omitted for the HETCOR-TOCSY, as these are identical to the HETCOR.

HMBC and COLOC provide similar ^n^J_CH_ (n = 2 or 3) correlations (supplementary material Fig. 2). Figure 2(b) compares anomeric expansions of the transposed HMBC with the COLOC experiments collected on laminarihexaose. Unlike HSQC, ^13^C decoupling is not used during the HMBC ^1^H acquisition, so the ^1^H resolution is higher for HMBC than HSQC. However, the ^13^C resolution in HMBC remains limited due to long acquisition times required to achieve high resolution. Like the HETCOR in Fig. 2(a), the ^13^C resolution in COLOC allows for more unambiguous assignments across the glycosidic linkages [Figs. 2(b) and 3].

Because the HMBC has greater overall sensitivity than COLOC, the HMBC spectrum shows additional correlations not observed in the COLOC experiment, including C_1_-H_5_ and C_1_-H_3_ intra-residue couplings [Fig. 2(b): HMBC peaks with no corresponding COLOC peak]. These peaks provide added confidence when validating residue specific assignments, so both HMBC and COLOC experiments were used to complete the assignments of both homohexamers in this study.

### C. HETCOR and COLOC structural assignment of polysaccharides

Correlations in the HETCOR provided ^1^J_CH_ pairs, serving as starting points for assignments. The HETCOR for laminarihexaose had 32 unique ^1^H–^13^C pairs (supplementary material Table IV), representing 3 fewer ^13^C chemical shifts than the 1D ^13^C (supplementary material Table III). The discrepancy in ^13^C chemical shifts was due to the high-resolution in the 1D ^13^C spectrum, which resolved shoulders of additional peaks compared to the HETCOR. Nevertheless, combining these ^1^H–^13^C pairs with the ^n^J_CH_ (n, >1) correlations in the COLOC enabled near complete assignments for all atoms of laminarihexaose (Table I, and supplementary material Fig. 5). The HETCOR-TOCSY resolved some of the more overlapped regions in the HETCOR alone, aiding in the detailed assignment. These datasets capture the α/β form of the reducing end at equilibrium, and residue ii is also sensitive to these two configurations. Similarly detailed assignments for sucrose (supplementary material Table V) and xylohexaose (supplementary material Table VI) are included in the supplemental materials.

**TABLE I.** Sequence and chemical shift assignment of laminarihexaose. Gray chemical shift assignments correspond to unresolved HETCOR peaks, though these regions contain multiple peaks in the 1D ^13^C. Question marks indicate some uncertainty in the sequence assignment at those glycosidic bonds, indicating iii and iv may be swapped. 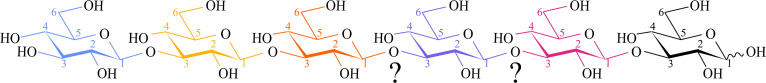

	β-D-Glc^vi^		β-D-Glc^v^		β-D-Glc^iv^		β-D-Glc^iii^		β-D-Glc^ii^ ^b^		β-D-Glc^i^ ^c^	
	^13^C	^1^H	^13^C	^1^H	^13^C	^1^H	^13^C	^1^H	^13^C	^1^H	^13^C	^1^H
**1**	105.529	4.746	105.234	4.790	105.253^a^	4.787^a^	105.253^a^	4.787^a^	105.266	4.766	98.398	4.666
**2**	76.161	3.353	75.955	3.551	76.012^a^	3.553^a^	76.012^a^	3.553^a^	76.012^a^	3.553^a^	76.550	3.430
**3**	78.261	3.518	86.946	3.772	86.770	3.781	86.770	3.781	86.770	3.781	87.198	3.739
**4**	72.288	3.406	70.831	3.515	70.797^a^	3.521^a^	70.797^a^	3.521^a^	70.797^a^	3.521^a^	70.831	3.515
**5**	78.714	3.478	78.332	3.499	78.332	3.499	78.332	3.499	78.332	3.499	78.291	3.482
**6**	63.409	3.713	63.389	3.734	63.389	3.734	63.389	3.734	63.389	3.734	63.420	3.729
**6**		3.913		3.920		3.920		3.920		3.920		3.889

^a^ Overlapping assignments across residues where there were single peaks in the HETCOR and 1D ^13^C.

^b^ Assignments for β-D-Glc^ii^ with α-D-Glc^i^ configuration: atom 1 (105.359 ^13^C ppm and 4.746 ^1^H ppm), atom 2 (76.034 ^13^C ppm and 3.557 ^1^H ppm), atom 3 (86.836 ^13^C ppm and 3.783 ^1^H ppm), atom 4 (70.797 ^13^C ppm and 3.521 ^1^H ppm), and atom 5 (78.332 ^13^C ppm and 3.499 ^1^H ppm).

^c^ Assignments of α-D-Glc^i^: atom 1 (94.733 ^13^C ppm and 5.228 ^1^H ppm), 3 (84.979 ^13^C ppm and 3.909 ^1^H ppm), and 5 (73.936 ^13^C ppm and 3.859 ^1^H ppm).

In both the homohexamers, there remained different degrees of uncertainty for the assigned sequence, which was depicted by question marks at the glycosidic bonds in question. For xylohexaose, the uncertainty was due in part to a greater degree of overlap for the anomeric carbons, although the long-range couplings for this hexamer also seemed more sensitive to the delay used for the COLOC, presenting fewer correlations than expected. For laminarihexaose, residue iii and iv share the same C1 ^13^C chemical shift, making it difficult to discern the β-D-Glc^iv^ (1 → 4) β-D-Glc^iii^ (1 → 4) β-D-Glc^ii^ correlations. However, the correlations observed between β-D-Glc^v^ (1 → 4) β-D-Glc^iv^ and β-D-Glc^ii^ (1 → 4) β-D-Glc^i^ leave only this sequence as a possibility.

## IV. DISCUSSION

NMR instrumentation has significantly improved over the past several years, especially with field strengths greater than 1 GHz and specialized cryogenic probes for ^13^C detection. The combination of these two technical advances enables the efficient measurement of ^13^C at natural abundance. With our 1.1 GHz instrument equipped with a ^13^C-optimized TXO cryoprobe, we were able to resolve nearly all the ^13^C resonances in sucrose and two different homohexameric polysaccharides: laminarihexaose and xylohexaose. Among the reported shifts (supplementary material Table III), subtle peak differences as low as 0.008 ppm for laminarihexaose were measured. This would not be possible at lower field strengths, especially with ^1^H detection.

Although there are databases and empirical chemical shift calculators for carbohydrate chemical shifts, these are limited by the existing data. For example, CASPER provided useful preliminary guidance for the two hexamers in this study, qualitatively predicting distinct chemical shifts for both terminal residues (see the supplementary material). However, for both the laminarihexaose and xylohexaose, the middle 4 residues were predicted to be identical, while the experimental 1D ^13^C spectra clearly showed that they are not. Supplementary material Figs. 6 and 7 show the difference between CASPER and experimental chemical shifts. Much of the difference can be explained by referencing, but smaller differences indicate subtle chemical shift changes between internal residues.

At high fields and with high-sensitivity ^13^C probes, HETCOR provides advantages in signal resolution over HSQC for single-bond correlations in these types of polysaccharides. The HETCOR-TOCSY sequence adds additional proton correlations within a residue. The COLOC experiment is then the analogous substitute for HMBC, which enables long-range correlations between carbons and protons across the glycosidic bond, thus linking the sugar residues. As noted in the Introduction, COLOC has not been widely used in the past two decades. As such, the original COLOC pulse sequence that we used in this study has not been optimized with modern shaped pulses and gradients, utilizing rectangular pulses with a 128 step phase cycle. At 28.5 T, a 200 ppm chemical shift range of ^13^C is about 55 kHz, which is impossible to cover with standard rectangular 180° ^13^C pulses. However, we were able to use the existing COLOC sequence because the ^13^C chemical shift range of the polysaccharides in this study was only about 12 kHz. We are confident that a modern update of COLOC, with shaped pulses and gradients to reduce the phase cycle, will produce better results and will significantly expand the ^13^C bandwidth to cover other classes of compounds.

Despite these limitations, we were able to largely assign laminarihexaose and sucrose and partially assign xylohexaose. The manual assignments of each of these polysaccharides is tedious and will not efficiently scale to the hundreds of structures that are relevant to the complex carbohydrates that comprise plant cell walls. The most significant observation from this study is that nearly all the 1D ^13^C NMR peaks were resolved in these challenging samples. This suggests a path forward in which entire libraries of known synthetic or biosynthetic polysaccharides of varying length could be measured by high-field 1D ^13^C and ^1^H NMR under automation and could be expanded to more complex branched glycans. Furthermore, experimental datasets from known samples could be the training data for a deep-learning model. Such a model would predict structures of unknown polysaccharides based on simple experimental NMR data. Select compounds could be manually analyzed by the suite of ^13^C observed 2D NMR experiments used in this study to validate model predictions.

The GeqShift model based on E(3)-equivariant graph neural networks^31^ and other recent 3D-based molecular representation learning (MRL) approaches^32^ exemplify current machine-learning methods aimed at improving carbohydrate chemical shift prediction and structural analysis. As these models continue to advance, their efficacy is increasingly limited by the availability of high-quality experimental data, particularly oligosaccharides, to benchmark their training. As neural networks for NMR analysis begin expanding, there is increasing motivation to co‐design experiments that are optimized for machine learning rather than solely for Fourier reconstruction. This philosophy was just recently demonstrated for studying protein dynamics and interactions, showcasing that information‐dense, time‐domain‐focused acquisitions processed by a deep neural network can recover high‐quality heteronuclear correlations in densely overlapped regions,^33^ a framework that could extend to polysaccharide assignments. By standardizing the measurements and providing all the time-domain NMR data in a public database, such as NAN or the BMRB, the experimental data will have more value and will ultimately lead to more robust and reliable ML/AI models in the future.

## V. CONCLUSION

A visionary sentiment was expressed three decades ago by Barbara Mulloy in a review titled *“High-field NMR as a technique for the determination of polysaccharide structures*,*”*^34^ which predicted that advances in magnetic resonance technology would increasingly enable detailed structural characterization of polysaccharides. Subsequent developments in NMR instrumentation have continued to affirm this insight, with more recent commentary similarly emphasizing that the rapid expansion of ultrahigh field spectrometers equipped with high sensitivity cryogenic probes will further accelerate the application of NMR spectroscopy to the analysis of these ubiquitous biomolecules.^35^ Indeed, recent demonstrations at magnetic field strengths of 1.0–1.5 GHz illustrate that ultrahigh‐field NMR now delivers outstanding spectral dispersion for both solid- and solution-state instruments. Nascent applications in large proteins, heterogeneous assemblies, and polysaccharides with repetitive internal residues demonstrate how previously intractable biomolecular systems and materials science are becoming accessible through enhanced spectral dispersion alone.^5,36,37^ Ever expanding, approaches can now capitalize on AI-guided ML algorithms that can resolve subtle spectral differences that previously lay beyond the reach of manual assignment.^33^ As a result, the structurally rich and chemically diverse field of carbohydrate research is poised for significant transformation. Deceptively simple in composition yet universal across all kingdoms of life, polysaccharides serve vastly diverse and untapped functions. Their importance, long recognized, is now more equally matched by the analytical power required to interrogate them at the level of individual residues and linkages using NMR.

## SUPPLEMENTARY MATERIAL

See the supplementary material for the following: detailed NMR acquisition and processing parameters (supplementary material Tables 1 and 2); ^13^C chemical shifts for sucrose, laminarihexaose, and xylohexaose (supplementary material Table 3); ^1^H–^13^C chemical shift pairs for sucrose, laminarihexaose, and xylohexaose (supplementary material Table 4); assigned chemical shifts for sucrose (supplementary material Table 5); assigned chemical shifts for xylohexaose (supplementary material Table 6); 1D ^1^H and ^13^C spectra of sucrose and xylohexaose (supplementary material Fig. 1); F1 and F2 dimensions for HSQC, HMBC, HETCOR, and COLOC (supplementary material Fig. 2); HSQC vs HETCOR comparison of xylohexaose (supplementary material Fig. 3); standard vs high-resolution HMBC for laminarihexaose (supplementary material Fig. 4); complete assignments of laminarihexaose with COLOC, HETCOR, and HMBC data (supplementary material Fig. 5); differences between experimental 1D and CASPER-predicted ^13^C chemical shifts for laminarihexaose and xylohexaose (supplementary material Fig. 6); differences between experimental HETCOR and CASPER-predicted ^1^H and ^13^C chemical shifts for laminarihexaose and xylohexaose (supplementary material Fig. 7); CASPER-predicted report for ^1^H and ^13^C chemical shifts for laminarihexaose; and CASPER-predicted report for ^1^H and ^13^C chemical shifts for xylohexaose.

## Data Availability

The data that support the findings of this study are openly available in the NAN Dashboard at https://usnan.nmrhub.org/data-browser/datasets, Ref. 38.
